# Subcutaneous immunoglobulin replacement for treatment of humoral immune dysfunction in patients with chronic lymphocytic leukemia

**DOI:** 10.1371/journal.pone.0258529

**Published:** 2021-10-15

**Authors:** S. Shahzad Mustafa, Saad Jamshed, Karthik Vadamalai, Allison Ramsey

**Affiliations:** 1 Division of Allergy, Immunology, Rheumatology, Rochester Regional Health, Rochester, New York, United States of America; 2 Division of Allergy, Immunology, Rheumatology, University of Rochester School of Medicine & Dentistry, Rochester, New York, United States of America; 3 Division of Hematology and Oncology, Rochester Regional Health, Rochester, New York, United States of America; 4 Division of Critical Care, Mercy Hospital, Springfield, Missouri, United States of America; National Institute of Animal Biotechnology, INDIA

## Abstract

**Background:**

Patients with chronic lymphocytic leukemia (CLL) experience hypogammaglobinemia and non-neutropenic infections. In this exploratory proof of concept study, our objective was to determine the prevalence of humoral immunodeficiency in patients with CLL and serum IgG ≥ 400 mg/dL, and to evaluate the efficacy of subcutaneous immunoglobulin (SCIG) in this population.

**Patients and methods:**

Patients with CLL with serum IgG ≥ 400 mg/dL were evaluated for serum IgG, IgM, IgA, along with pre/post vaccine IgG titers to diphtheria, tetanus, and *Streptococcus pneumoniae*. Patients with evidence of humoral dysfunction were treated with SCIG with Hizentra every 7±2 days for 24 weeks.

**Results:**

Fifteen patients enrolled with median IgG = 782 mg/dL [IQR: 570 to 827], and 6/15 (40%) responded to vaccination with Td, while 5/15 (33%) responded to vaccination with PPV23. 14/15 (93.3%) demonstrated humoral immunodeficiency as evidenced by suboptimal vaccine responses, and were treated with SCIG. In patients treated with SCIG, serum IgG increased from 670 mg/dL [IQR: 565 to 819] to 1054 mg/dL [IQR: 1040 to 1166] after 24 weeks (95% CI: 271–540). For *streptococcus pneumoniae*, the median protective serotypes at baseline was 8 [IQR: 4 to 9] and increased to 17 [IQR: 17 to 19] after 24 weeks (95% CI: 6.93–13.72). Non-neutropenic infections (NNI) decreased from 14 to 5 during treatment with SCIG.

**Conclusions:**

Patients with CLL demonstrate humoral immunodeficiency despite IgG > 400 mg/dL. For these patients, SCIG is well tolerated and efficacious in improving serum IgG, specific IgG to *streptococcus pneumoniae*, and may decrease reliance on antibiotics for the treatment of NNIs.

**Clinical trials registration:**

NCT 03730129.

## Introduction

Chronic lymphocytic leukemia (CLL) is the most common adult leukemia with approximately 21,000 new patients diagnosed annually in the United States [1]. Patients with CLL are at increased risk of infections [2] and up to one-half of patients with CLL experience an infectious complication during their disease course, with up to 17% to 50% of these being fatal [3]. Infections are typically bacterial in nature and most commonly affect the sino-pulmonary tract. Hypogammaglobinemia is the primary cause of increased susceptibility to infections in CLL and is present in up to 85% of CLL patients. Hypogammaglobinemia is due to both disease progression and the use of anti-neoplastic agents [4]. CLL decreases the ability of B lymphocytes to produce immunoglobulins due to defective functioning of non-clonal CD5-negative B cells, and also increases apoptosis of normal immunoglobulin (Ig)-producing plasma cells [5]. Mutations in the immunoglobulin heavy chain variable region may also contribute to abnormal Ig levels, although there is conflicting data on the clinical consequences of these mutations. Additionally, abnormal function of B lymphocytes can also adversely affect cross-talk with T-helper cells, thus further decreasing an adequate immune response.

Strategies to decrease infectious complications in patients with CLL include routine vaccination, use of prophylactic antibiotics, and immunoglobulin replacement (IgR) therapy. Although no clinical studies have rigorously evaluate the use of prophylactic antibiotics, this strategy is commonly implemented and recommended for prophylaxis against infections for patients with CLL [6]. IgR has been studied for infection prophylaxis in CLL, and its use is universally supported in patients with CLL with hypogammaglobinemia and associated infections [7–13]. As demonstrated by the international survey by Na et al., roughly 30% of patients with CLL are treated with IgR at some point during their disease management, and this proportion is similar to other hematological malignancies such as multiple myeloma and non-Hodgkin’s lymphoma [14]. Nevertheless, there remains heterogeneity in practice of when clinicians initiate IgR therapy in CLL, and guidelines vary from country to country [15]. Although IgR has been shown to decrease rates of bacterial infection in some studies, the optimal application of this practice remains unclear. A Cochrane meta-analysis concluded that the use of intravenous IgR may be considered in patients with CLL and other lymphoproliferative diseases (LPD) who have hypogammaglobinemia and recurrent infection, but acknowledged that the studies are heterogeneous [16].

Previous studies have shown that patients with CLL have impaired humoral immunity as defined by vaccine responses to both polysaccharide (e.g. pneumococcal) and peptide (e.g. tetanus or diphtheria) antigens [16]. Patients with primary immunodeficiency are routinely evaluated for their humoral antibody response before IgR is considered. However, the practice of checking vaccine responses prior to starting immunoglobulin replacement has been advocated, but not extensively studied in patients with CLL [4, 17–19]. Data has shown that low titers to pneumococcal vaccination had a better association with risk of infection compared to serum IgG [20]. Thus, an immune evaluation including vaccine titers may help to identify CLL patients most at risk for life threatening infection as compared to patients with hypogammaglobinemia but preserved humoral immunity, or patients with clinical infections despite preserved immunoglobulin levels and function. Patients with impaired humoral immunity, regardless of Ig level, may benefit most from IgR.

Previous trials and clinical practice have primarily used intravenous immunoglobulin (IVIG) in patients with CLL with recurrent infections and hypogammaglobinemia [9, 13, 21]. As compared to subcutaneous immunoglobulin (SCIG), IVIG carries a higher risk of an infusion reaction, aseptic meningitis, renal dysfunction, hypercoagulability, and requires a trained medical personnel to provide intravenous access [22, 23]. SCIG mitigates these risks and can also more easily be administered at home, providing much greater patient autonomy [23–25]. There is limited data on the use of SCIG in patients with secondary immunodeficiency due to CLL, but one center has published experience with SCIG in 61 patients with LPD, including patients with CLL [26].

This study is novel because it stratified patients with CLL according to their humoral response to peptide and polysaccharide vaccines, and then utilized SCIG in those with an impaired humoral response.

## Patients and methods

Patients were referred for immunologic evaluation for this prospective case series from local hematology/oncology offices in Rochester, NY. All study procedures were conducted at the Rochester Regional Health allergy/immunology practice in Rochester, NY from March 2019 through June 2020. The authors confirm that all ongoing and related trials for this drug/intervention were registered prior to patient enrollment (NCT 03730129). The study was approved by the institutional review board at Rochester Regional Health on October 10, 2018 (CIC 1850-A-18), and all patients signed informed consent.

**Fig 1:**
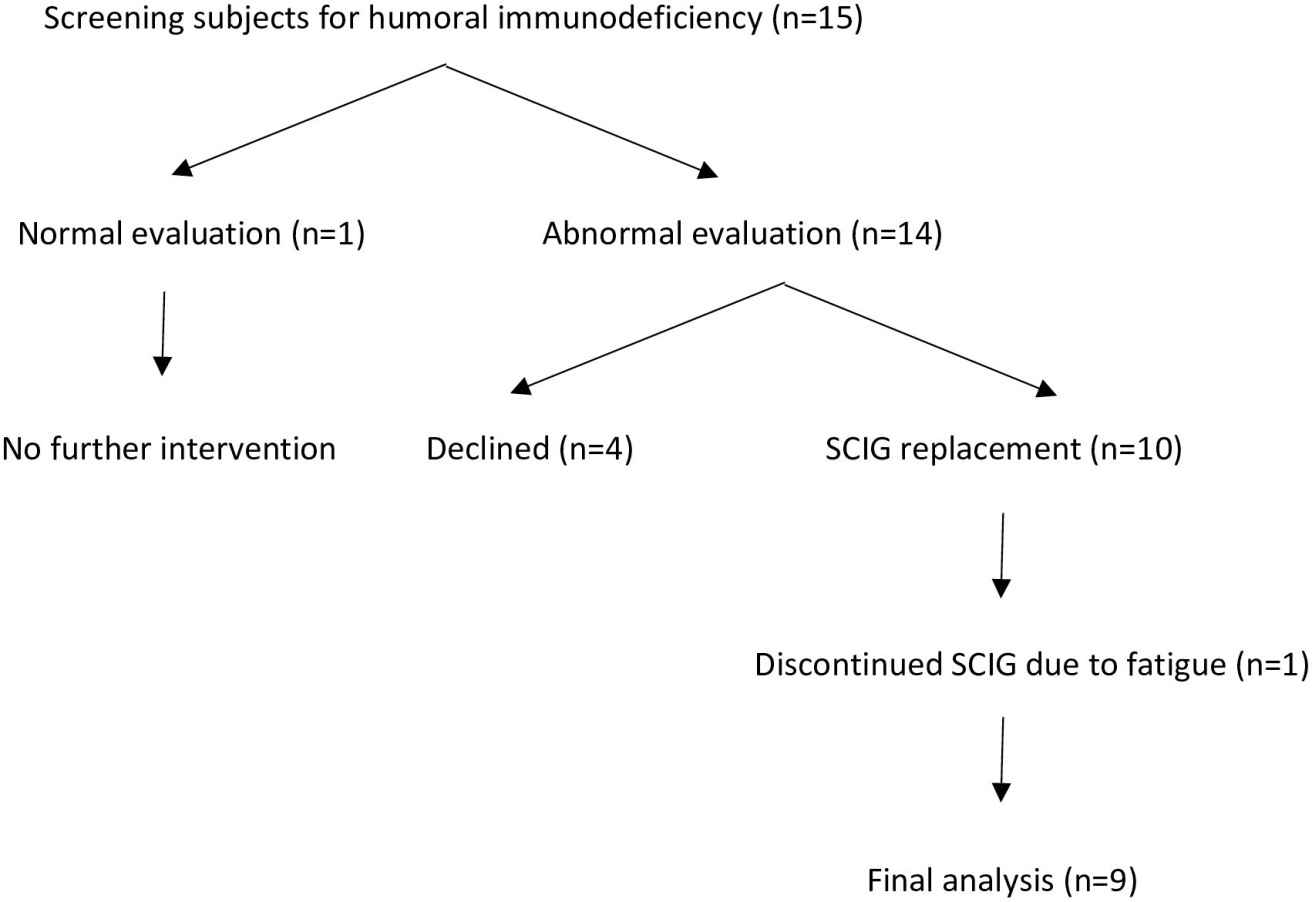
Study schematic.

Patients with a confirmed diagnosis of CLL per flow cytometry either on peripheral blood or bone marrow biopsy were eligible for enrollment in the study (Fig 1). Exclusion criteria included previously diagnosed primary immunodeficiency, additional immunosuppressive states as defined by the investigators, serum IgG < 400 mg/dL, and previous or ongoing therapy with IgR. Patients underwent a laboratory evaluation for immunodeficiency, including baseline serum IgG, IgM, and IgA, along with pre and post-vaccination IgG titers for peptide antigens (diphtheria, tetanus) with Td and polysaccharide antigens (*Streptococcus pneumoniae)* with pneumococcus polyvalent vaccine-23 (PPV23) 28±7 days following vaccination. Normal vaccine responses were defined by the following criteria [27]:

1. Diphtheria: 2-fold increase, and must be into protective range (0.1 IU/ml)
2. Tetanus: 2-fold increase, and must be in protective range (0.1 IU/ml)
3. Streptococcus pneumoniae
  If < 1.3 μg/ml, 2-fold increase to > 1.3 μg/ml OR 4-fold increase
  If > 1.3 μg/ml, 2-fold increase
  Responses must be demonstrated by 70% of serotypes
4. Impaired vaccine response is defined at abnormal response to any of the above antigens.

Patients with humoral immunodeficiency (as evidenced by an abnormal vaccine response) despite serum IgG ≥ 400 mg/dL were offered therapy with 20% liquid SCIG (Hizentra, CSL Behring, King of Prussia, PA) for 24 weeks regardless of infection history. SCIG was given at a fixed dose of 0.13 g/kg/week, and administered every 7±2 days. Patients did not receive a loading dose of immunoglobulin. Patients were not pre-medicated prior to SCIG therapy unless they experienced infusion-related side effects. Patients received instruction on SCIG administration for 3–4 sessions from a research coordinator, and then transitioned to independent administration at home. The number of infusion sites and time for infusion was tracked for each patient, as were adverse events. SCIG was provided by the sponsor, CSL Behring.

The following data was collected for each patient: demographic data (age, race, and gender), time since initial diagnosis of CLL, chemotherapeutic regimen and other therapies used for management of CLL, and number of non-neutropenic infections (NNI) requiring therapy with antibiotics in the previous 6 months (per patient recall in combination with a review of the electronic medical record). All patients underwent a laboratory evaluation for humoral immunodeficiency as defined above. For patients who were treated with SCIG the number of infusion sites and duration of infusion was tracked throughout the study period, as were adverse events. Patients on SCIG completed a quality of life survey (Short Form 36) every 28±7 days. Ig levels and IgG titers for diphtheria, tetanus, and streptococcus pneumoniae were checked 4, 12, and 24 weeks after starting IgR. A repeat evaluation for humoral immunodeficiency, including pre and post-vaccination IgG titers was completed 3 months after the last dose of SCIG.

### Statistical analysis

Statistical analysis was performed using STATA software (StataCorp LLC, College Station, Texas). Baseline characteristics along with both the primary and secondary endpoints are reported in medians and interquartile (IQR) ranges. A non-parametric, Wilcoxon sign ranked test was used to compare study outcomes at baseline and the completion of the study. Due to the exploratory nature of this proof of concept study, a formal power calculation was not completed prior to study initiation, and the study was not adequately powered to test a specific hypothesis. Microsoft Excel software (Office 365, Microsoft Corporation, Redmond, WA 98052) was used to create the figures.

## Results

A total of 18 patients underwent evaluation, with 15 enrolled in the study (Table 1). Three patients were excluded due to IgG < 400 mg/dL. There were 13 (86.7%) males and 2 (13.3%) females. The median age of the cohort was 69 years [IQR: 67 to 75]. The median time since diagnosis of CLL was 4.4 years [IQR: 2.6 to 15.2]. The median number of NNI treated with antibiotics was 1 [IQR: 0 to 2]. Previous to enrollment, four patients had undergone treatment regimens for CLL. At the time of the study, one patient was being treated with ibrutinib, while 14 of 15 patients were managed with close observation.

**Table 1:** Demographics.

Patient	Age	Sex	Time since diagnosis (years)	NNI treated with antibiotics (previous 6 months)	Current treatment	Previous treatment
1	68	M	14.6	5	Ibrutinib	Fludarabine + rituximab in 2011, rituximab in 2013, bendamustine + cyclophosphamide in 2014
2	75	F	8.3	3	None	None
3	76	M	18.4	1	None	None
4	68	M	20.4	2	None	None
5	66	F	1.8	0	None	None
6	68	M	15.8	0	None	Radiation to tonsil bed
7	71	M	0.5	2	None	None
8	70	M	23.0	0	None	Fludarabine 2003–1013, fludarabine + cyclophosphamide + rituximab 2012–2013
9	56	M	1.8	0	None	None
10	69	M	0.3	1	None	None
11	75	M	3.4	0	None	None
12	79	M	4.3	1	None	None
13	62	M	4.4	2	None	None
14	86	M	4.3	0	None	None
15	53	M	4.7	0	None	Vincristine + cyclophosphamide + rituximab 2012–2013, bendamustine + rituximab 2014–2015, ublituximab + umbralisib 2019

NNI: non-neutropenic infection.

The baseline immune evaluation was as follows: median IgG = 782 mg/dL [IQR: 570 to 827], median IgM = 44 mg/dL [IQR: 37 to 68], and median IgA = 138 mg/dL [IQR: 81 to 171] (Table 2). At enrollment, the median IgG for tetanus = 1.21 IU/ml [IQR: 0.85 to 1.50] increased to a median of 2.03 IU/ml [IQR: 1.01 to 2.76] following vaccination. Six of the 15 (40%) patients responded to vaccination with tetanus. The median IgG for diphtheria = 0.16 IU/ml [IQR: 0.05 to 0.27] decreased to a median of 0.14 IU/ml [IQR: 0.1 to 0.34] following vaccination. Six of the 15 (40%) patients responded to vaccination with diphtheria. For baseline IgG for streptococcus pneumoniae, the median number of serotypes with protective titers of ≥ 1.3 mcg/ml was 9/23 [IQR: 5 to 12], and this changed to 9/23 [IQR: 6 to 17] following vaccination. Five of the 15 (33%) patients responded to vaccination with PPV23.

**Table 2:** Ig levels and vaccine responses.

				Tetanus IgG, (IU/mL)			Diphtheria IgG, (IU/ml)			Streptococcus Pneumoniae Protective Serotypes (≥ 1.3 mcg/ml)			
Patient	IgG, (mg/dL)	IgM, (mg/dL)	IgA, (mg/dL)	Pre-vaccine	Post- vaccine	Responder	Pre-vaccine	Post-vaccine	Responder	Pre-vaccine	Post-vaccine	Responder	Subq IgR Enrollment
1	559	48	98	0.72	0.61	No	0.05	0.04	No	10	9	No	Yes
2	443	45	65	1.21	2.98	Yes	0.11	0.34	Yes	7	11	No	Yes
3	419	16	43	0.40	0.41	No	0.02	0.03	No	2	2	No	Yes
4	684	41	167	1.79	2.03	No	0.16	0.14	No	9	7	No	Yes
5	831	44	100	1.54	4.05	Yes	0.58	>3.00	Yes	8	8	No	Yes
6	977	44	175	0.98	1.09	No	0.1	0.12	No	5	7	No	Yes
7	581	12	38	0.50	2.34	Yes	0.16	0.13	No	2	2	No	Yes
8	655	120	149	1.12	0.81	No	0.2	0.19	No	9	17	Yes	Yes
9	823	79	151	1.85	6.21	Yes	0.27	1.03	Yes	3	4	No	Yes
10	808	76	225	1.07	0.94	No	0.51	0.52	No	14	14	No	Yes
11	941	60	200	1.47	1.34	No	0.44	0.92	Yes	19	20	Yes	Declined
12	788	228	254	0.10	1.24	Yes	0.02	0.14	Yes	13	16	Yes	Not eligible
13	494	30	57	1.88	2.35	No	<0.01	0.09	No	10	17	Yes	Declined
14	1039	42	138	1.41	6.12	Yes	<0.01	<0.01	No	18	21	Yes	Declined
15	782	32	97	1.35	2.54	No	0.05	0.1	Yes	5	3	No	Declined
Median	782	44	138	1.21	2.03	6/15	0.16	0.14	6/15	9	9	5/15	
IQR	(570–827)	(37–68)	(81–171)	(0.85–1.50)	(1.01–2.76)	(40%)	(0.05–0.27)	(0.1–0.34)	(40%)	(5–12)	(6–17)	(33%)	

Of the 15 patients in the cohort, 14 (93.3%) met criteria for humoral immunodeficiency due to poor vaccine responses to peptides and/or polysaccharides, and were offered therapy with SCIG. One patient declined due to deterioration in clinical status and enrollment into an alternative clinical study. Three patients declined because they did not wish to pursue IgR given their currently stable health status. A total of 10 agreed to pursue therapy, with 9 completing 24 weeks of therapy. One patient discontinued IgR early due to reported fatigue (Table 3). The median weekly dose was 11.5 g [IQR: 11 to 12], with each infusion utilizing a median of 2.5 sites, and taking 61 minutes [IQR: 56 to 64]. Nine of the 10 patients did not require pre-treatment with antihistamines, acetaminophen, NSAIDs, or systemic steroids prior to SCIG infusions. There were no localized or systemic reactions. One patient reported fatigue for 2–3 days after SCIG infusions and was pre-treated with diphenhydramine and acetaminophen with no improvement of symptoms. This patient subsequently discontinued therapy with SCIG.

**Table 3:** Dosing and adverse events related to subq IgR in study population.

Patient	Weight (kg)	Weekly Dose (g)	Weekly Dose (g/kg/week)	# of sites (average)	Infusion time (min) (average)	Pre-medication regimen	Adverse events
1	91	12	0.133	2.5	55	None	None
2	78	10	0.129	2.5	64	None	None
3	88	12	0.137	3	56	None	None
4	103	12	0.117	2.5	57	None	None
5	84	11	0.131	2.5	63	None	None
6	137	18	0.132	3	64	None	None
7	85	11	0.130	2.5	64	None	None
8	104	13	0.126	2.5	56	Diphenhydramine, acetaminophen	Fatigue
9	88	11	0.125	2	62	None	None
10	86	11	0.127	2	59	None	None
Median	88	11.5	0.12	2.5	61		
IQR	(85–100)	(11–12)	(0.12–0.13)		(56–64)		

**Fig 2:**
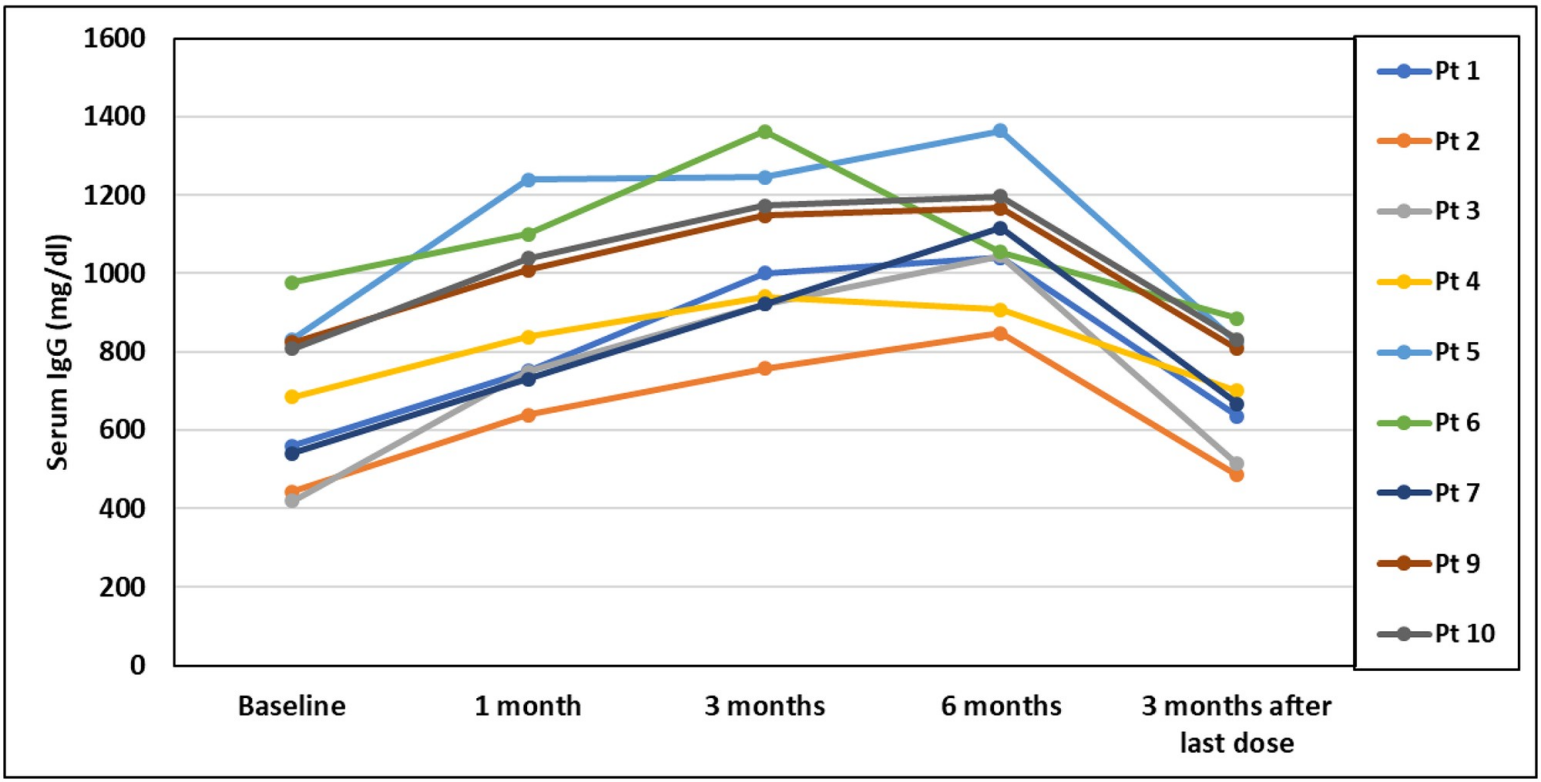
Serum IgG levels.

**Fig 3:**
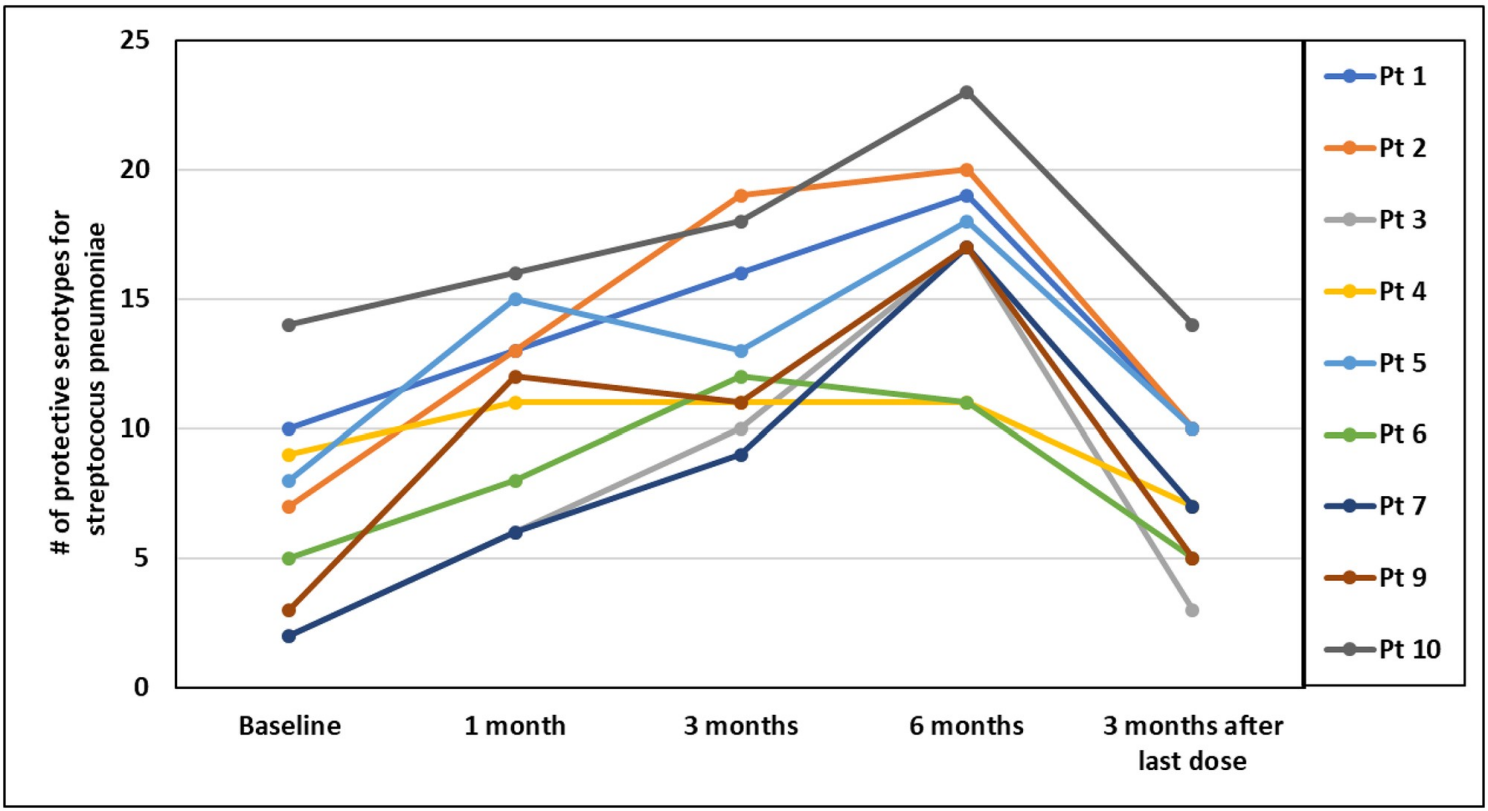
Number of protective serotypes for *streptococcus pneumonia*.

In the 9 patients who completed 24 weeks of IgR, the median baseline IgG of 670 mg/dL [IQR: 565 to 819] increased to 838 mg/dL [IQR: 749 to 1038] after 4 weeks (95% CI: 154–291), 1001 mg/dL [IQR: 922 to 1173] after 12 weeks (95% CI: 319–432), and 1054 mg/dL [IQR: 1040 to 1166] after 24 weeks (95% CI: 271–540) (Fig 2). Three months after discontinuation of IgR, IgG = 701 mg/dL [IQR: 635 to 823], which was similar to baseline (95% CI: 21–88). For tetanus, the median baseline IgG of 1.07 IU/ml [IQR: 0.72 to 1.54] increased to 1.93 IU/mL [IQR: 1.29 to 3.00] after 4 weeks (95% CI: 0.42–2.17), 2.56 IU/mL [IQR: 2.23 to 4.47] after 12 weeks (95% CI: 0.94–3.67), and 2.14 IU/mL [IQR: 1.93 to 2.62] after 24 weeks (95% CI: 0.8–1.6). Three months after discontinuation of IgR, IgG for tetanus was 1.54 IU/mL [IQR: 1.04 to 1.81], which was still higher than baseline (95% CI: 0.19–1.08). For diphtheria, the median baseline IgG of 0.16 IU/ml [IQR: 0.10 to 0.27] increased to 0.27 IU/mL [IQR: 0.18 to 0.60] after 4 weeks (95% CI: -0.17–1.0), 0.35 IU/mL [IQR: 0.27 to 0.99] after 12 weeks (95% CI: -0.03–1.57), and 0.41 IU/mL [IQR: 0.31 to 0.69] after 24 weeks (95% CI: -0.01–1.09). Three months after discontinuation of IgR, IgG for diphtheria was 0.23 IU/mL [IQR: 0.19 to 0.42], which was still higher than baseline (95% CI: -0.08–0.59). For *streptococcus pneumoniae*, the median number of serotypes in the protective range (> 1.3 mcg/ml) at baseline was 8 [IQR: 4 to 9] and increased to 13 [IQR: 9 to 15] after 4 weeks (95% CI: 2.59–6.29), 12 [IQR: 11 to 16] after 12 weeks (95% CI: 4.37–8.73), and 17 [IQR: 17 to 19] after 24 weeks (95% CI: 6.93–13.72) (Fig 3). Three months after discontinuation of IgR, protective serotypes for *streptococcus pneumoniae* = 7 [IQR: 5 to 10], which was similar to baseline (95% CI: 0.39–2.94).

**Fig 4:**
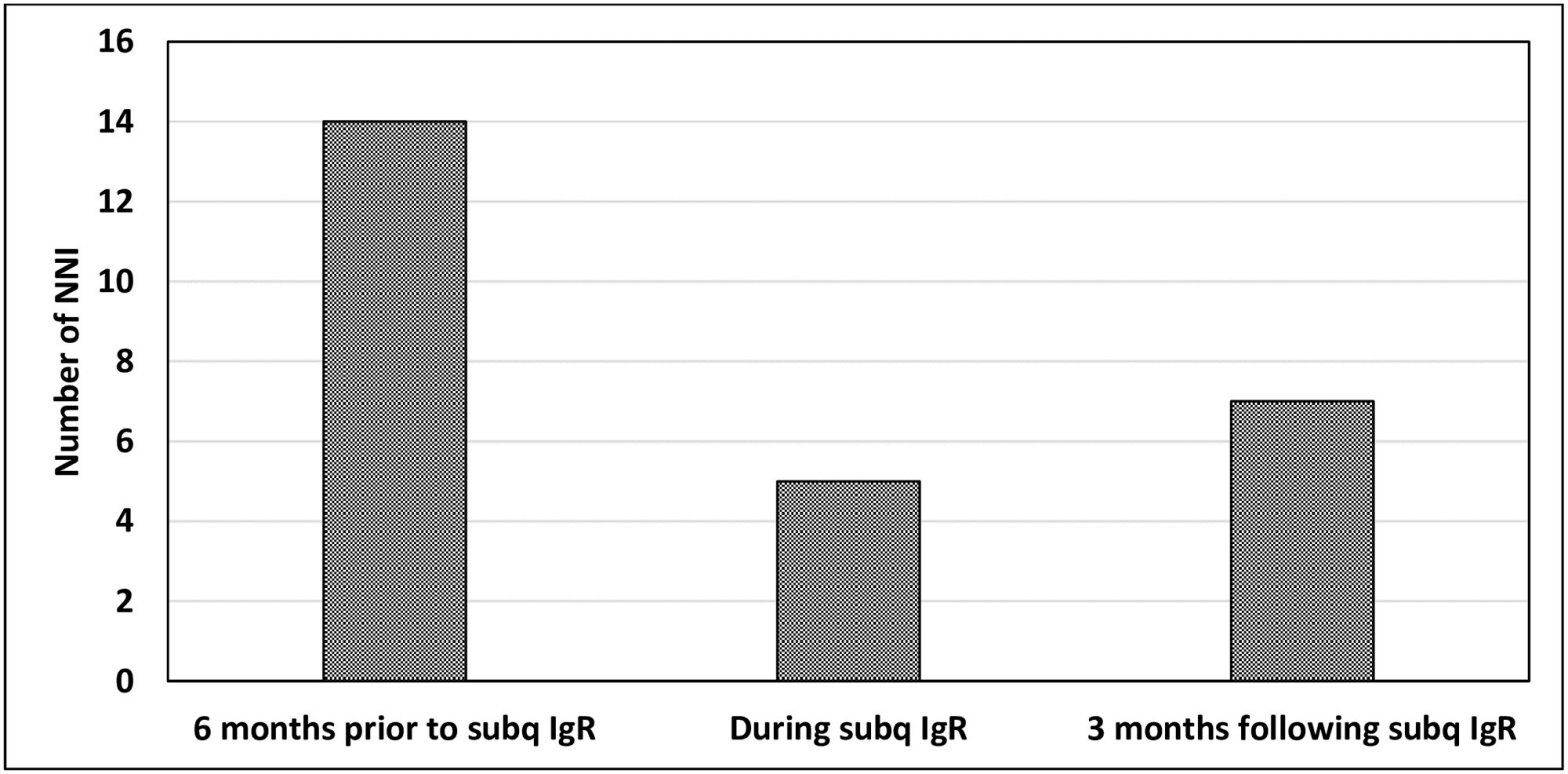
NNI prior to, during subq IgR, and after subq IgR. NNI: non-neutropenic infection, subq: subcutaneous, IgR: immunoglobulin replacement.

The 9 patients who completed 24 weeks of IgR experienced 14 NNI in the 6 months prior to IgR (one requiring inpatient hospitalization), and this decreased to 5 NNI during 6 months of therapy with IgR, with no hospitalizations (Fig 4). Additionally, within the 3 months following IgR, these patients experienced 7 NNI. There was no significant change in the Short Form 36 values during the study period.

## Discussion

Patients with CLL are recognized to be at increased risk of infections due to a myriad of factors, including hypogammaglobinemia [2, 4], and infections remain a major cause of morbidity and mortality in this population [3]. Although IgR has been used as a strategy for infection prophylaxis [7–13], our study is unique because it proactively completed an immune evaluation by checking vaccine responses to peptide and polysaccharide antigens in patients with CLL regardless of infection history. We demonstrated that patients with CLL are at risk of humoral dysfunction despite relatively normal Ig levels. Furthermore, prophylactic therapy with SCIG increased serum IgG and specific antibody titers, was well tolerated, and showed a trend in decreased NNIs.

There are no consensus guidelines on the evaluation of humoral immunity in patients with CLL. Although the risk of infection in CLL is felt to generally increase with lower IgG levels [28], not all patients with hypogammaglobinemia will experience infectious complications [29]. In contrast, there is a subset of individuals with CLL who experience increased infectious episodes despite having normal or near-normal IgG levels, due to B lymphocytes producing non-functional, monoclonal Ig [30–32]. Significant B cell dysfunction likely also arises from aberrant signaling from the B cell receptor (BCR), which is characterized by variable response to antigenic stimulation, thus affecting other immune functions, including T cell and cellular immunity. Due to the possibility of abnormal functional status despite relatively normal IgG levels, and based on our study results, we encourage the evaluation of vaccine responses to peptide and polysaccharide antigens to help determine the function of Ig in CLL patients. Our cohort of CLL patients all with serum IgG > 400 mg/dL, and a median serum IgG of 782 mg/dl [IQR: 570 to 827] showed only 1/15 patients (6.7%) mounted an adequate immune response to vaccination with Td and PPV23. Our findings are in line with previous research showing similarly poor vaccine responses in this population [33–36], and highlight the importance of evaluating vaccine responses for a thorough immune evaluation in patients with CLL. Early identification and treatment of secondary immune deficiency may improve morbidity and possibly mortality in these patients. Additionally, three patients (16.7%) who underwent an immune evaluation demonstrated IgG < 400 mg/dl, with one patient having an IgG = 93 mg/dL. We therefore believe risk stratification based on this immune evaluation should be routinely considered in all patients with CLL regardless of previous infection history, since this may reveal severe immune dysfunction, even early in disease.

Immunoglobulin replacement provides passive immunity by providing a diverse antibody repertoire from healthy donors, and is commonly used in CLL for infection prophylaxis. Although IgR is not felt to be immune modulating, with no direct effects on immune dysfunction, replacement of functional antibodies has been shown to decrease infectious complications [26]. We are among the first to show the potential utility of SCIG in CLL patients with secondary immune deficiency, as the majority of CLL patients treated with IgR receive intravenous infusions. Similar to Compagno et al [26], 90% of patients in our cohort reported no adverse effects related to SCIG infusions. Nine of ten patients also did not require pre-medication, and the majority completed infusions independent of medical professionals in one hour while at home. Self-administered at home administration is particularly valuable during the current global pandemic with COVID-19 infection since it allows for minimizing unnecessary interaction with health care facilities. We encourage all clinicians prescribing IgR to engage in shared decision making with their patients regarding the most suited route of IgR. This discussion is also a unique opportunity for collaboration between hematology/oncology and allergy/immunology colleagues, since the latter utilize routinely utilize SCIG in the management of primary immunodeficiency.

Our results also show promising efficacy of SCIG in the setting of CLL. All of the patients in our cohort experienced significant increases in their IgG levels, and this was significant as early as four weeks after the initiation of IgR. In addition to an increase in serum IgG, our study is the first to show a significant improvement in specific IgG for peptide antigens such as tetanus and diphtheria, and polysaccharide antigens such as *Streptococcus pneumonia*. This is likely to be particularly meaningful, since *Streptococcus pneumoniae* is felt to be one of the most common etiologies of recurrent sino-pulmonary infections in patients with CLL [37]. Most importantly, for our cohort of patients with CLL, these improvement in laboratory findings while on SCIG correlated with a decreased reliance on antibiotics for the treatment of NNI. Although our results did not show a significant change in health-related quality of life scores, we suspect this is because our enrollment criteria did not require a previous history of infection, and many patients reported a high quality of life at enrollment despite an abnormal immune evaluation.

We acknowledge limitations to our study. Most importantly, our study was small in size, and did not require a history of NNIs for enrollment. We suspect a larger cohort with a greater history of NNIs requiring frequent antibiotic therapy would benefit even more from our diagnostic and treatment strategy. Due to our small sample size, we were not able to formally evaluate for improvement in NNIs. Importantly, although patients served as their own control, our study also did not have a control arm, which makes it more difficult to assess the clinical impact of IgR in this patient population. Future, larger studies are warranted to evaluate the efficacy of risk stratification with vaccine response, followed by treatment with SCIG in patients with an abnormal evaluation. We also did not evaluate the potential role of conjugate pneumococcal vaccination (PCV13) as a strategy to improve *Streptococcus pneumoniae* titers in patients not responding to polysaccharide vaccination (PPV23), as this may serve as adequate protection in a subset of individuals and may avoid the need for IgR. Despite these limitations, we believe our study is novel in its use of vaccine responses to evaluate for humoral immunodeficiency, and provides foundational data for the use of SCIG in the setting of CLL despite adequate IgG levels.

In conclusion, this study suggests that patients with CLL demonstrate significant humoral immunodeficiency as defined by abnormal vaccine responses even in the setting of relatively normal IgG levels (> 400 mg/dL). For these patients, SCIG is likely to be well tolerated and efficacious in improving serum IgG, specific IgG to *streptococcus pneumoniae*, and may decrease reliance on antibiotics for the treatment of NNIs. Larger studies using vaccine responses as risk stratification for infectious complications are needed, as are randomized controlled trials of subq IgR in patients with CLL.

## Supporting information

S1 Checklist (PDF)

S1 File (XLSX)

S2 File Study protocol. (DOCX)

S3 File (DOCX)
