# Supplementary material for: Subcutaneous immunoglobulin replacement for treatment of humoral immune dysfunction in patients with chronic lymphocytic leukemia
Source: PLoS One. 2021 Oct 15;16(10):e0258529. doi: 10.1371/journal.pone.0258529 (PMC8519417; doi:10.1371/journal.pone.0258529)
Supplement: S2 File — (DOCX) [file pone.0258529.s003.docx]

**1**

**A prospective case series of subcutaneous immunoglobulin for prophylaxis of infections**

**in patients with chronic lymphocytic leukemia with impaired humoral immunity**

Principle investigator: S Shahzad Mustafa, MD

Co-investigators: Allison Ramsey, MD, Saad Jamshed, MD

Sponsor: The Rochester General Hospital

Funding and drug will be provided via a grant from CSL Behring.

March 27, 2019

**1. Protocol Summary**

**1.1 Synopsis**

**Title:**

A prospective case series of subcutaneous immunoglobulin for prophylaxis of infections in patients with chronic lymphocytic leukemia with impaired humoral immunity

**Study Description:**

Patients with chronic lymphocytic leukemia (CLL) are at increased risk of infections as compared to age matched controls, with infections being a major cause of morbidity and mortality. Previous studies have shown that patients with CLL have both hypogammaglobinemia and impaired humoral immunity as defined by vaccine responses to both polysaccharide and peptide antigens. Attempts at decreasing infections in CLL have included therapy with prophylactic antibiotics and intravenous immunoglobulin. In general clinical practice and in previous studies, patients have started IV immunoglobulin replacement therapy if they have a history of serious infection or hypogammaglobinemia (defined as IgG below 400 g/dL), but vaccine responses have not been evaluated. This study will identify CLL patients with humoral immunodeficiency by checking both Ig levels and vaccines responses. In patients with impaired humoral immunity, we will use subcutaneous immunoglobulin replacement to show this intervention will increase Ig levels, protective antibody titers, and be well tolerated.

**Objectives:**

*Primary objective:*

To identify patients with CLL with humoral immunodeficiency despite serum IgG ≥ 400 mg/dL

*Secondary objectives:*

1. Efficacy of subcutaneous Ig replacement therapy in increasing Ig levels

2. Efficacy of subcutaneous Ig replacement therapy in increasing protective antibody titers for diphtheria, tetanus, streptococcus pneumoniae

3. Safety and tolerability of subcutaneous Ig replacement therapy

4. Quality of life (SF 36) on subcutaneous Ig replacement therapy

5. Track the number of infections requiring antibiotics, further characterized per severity as defined in previous studies.

**Endpoints:**

1. Identify patients with CLL with humoral immunodeficiency despite serum IgG ≥400 mg/dL.

2. Demonstrate efficacy of subcutaneous Ig replacement therapy in increasing Ig levels and protective antibody titers.

**Study Population:**

*Sample size:*

From previous literature, we expect to evaluate 20-30 patients for secondary immunodeficiency to reach a goal of 10 patients to undergo treatment with subcutaneous immunotherapy.

*Gender:*

Both males and females will be enrolled in this study.

*Age:*

Eligible patients will be ages 18 and above.

*General health status:*

All patients with CLL who are medically stable with an expected survival of > 1 year per their primary hematologist/oncologist will be offered enrollment in this study.

*Geographic area:*

Patients will be recruited from hematology/oncology practices in Rochester, NY. The majority of patients will come from the CLL Registry and Rochester Regional Health, which includes over 300 patients. Other local hematology/oncology practices may also contribute patients to the study.

**Phase:**

Phase 2

**Description of Sites and Enrollment:**

This is a single center study. Patients will be recruited from hematology/oncology practices in Rochester, NY. The majority of patients will come from the CLL Registry and Rochester Regional Health, which includes over 300 patients. Other local hematology/oncology practices may also contribute patients to the study. All patients will be evaluated at the allergy/immunology office at Rochester Regional Health.

**Description of Study Intervention:**

Patients with humoral immunodeficiency will receive subcutaneous immunoglobulin replacement (Hizentra 100 mg/kg/week).

**Study Duration:**

We expect to enroll 10 subjects for the therapy phase of the study within 1 year. Each subject will undergo evaluation for humoral immunodeficiency over a 4-week period. Patients with abnormal evaluation will be treated with 6 months (24 weeks) of subcutaneous Ig replacement therapy, followed by a 3 month (12 week) wash out period, for a total study duration of 10 months (40 weeks). We plan to complete all study visits and analyze data within two years of when enrollment starts.

**Participant Duration:**

The entirety of the study will entail 10 months (40 weeks) for each subject:

4 weeks of laboratory evaluation

24 weeks of subcutaneous Ig replacement

12 weeks of wash out period

**1.2 Schema**

Please see below.

Screening subjects for humoral immunodeficiency (n = 20-30)

4 weeks

Normal evaluation (n = 10-20) Abnormal evaluation (n=10)

No further intervention Subcutaneous Ig replacement

24 weeks weeks

Discontinue Ig replacement

12 weeks

Final lab evaluation

**1.3 Schedule of Activities**

Please see below.

|  | Screening | | Therapy with Ig Replacement | | | | | | | | | Wash Out Period | | | |
| --- | --- | --- | --- | --- | --- | --- | --- | --- | --- | --- | --- | --- | --- | --- | --- |
| Study visit | V1 |  | V2 | V3 | V4 | V5 | V6 | V7 | V8 | V9 | V10 | V11 | V12 | V13 | V14 |
| Week of study |  |  | 1 | 2 | 3 | 4 | 8 | 12 | 16 | 20 | 24 | 28 | 32 | 36 | 40 |
| Consent | X |  |  |  |  |  |  |  |  |  |  |  |  |  |  |
| Urine pregnancy test* | X |  |  |  |  |  |  |  |  |  |  |  |  |  |  |
| Medical history |  |  | X | X | X | X | X | X | X | X | X | X | X | X | X |
| Vitals |  |  | X | X | X | X | X | X | X | X | X | X | X | X | X |
| Physical exam |  |  | X | X | X | X | X | X | X | X | X | X | X | X | X |
| QoL (SF 36 form) |  |  | X | X | X | X | X | X | X | X | X | X | X | X | X |
| Pt teaching for Ig |  |  | X | X | X |  |  |  |  |  |  |  |  |  |  |
| Dispense study Ig |  |  |  |  |  | X | X | X | X | X | X |  |  |  |  |
| Pneumovax (PPV23) | X |  |  |  |  |  |  |  |  |  |  |  |  | X |  |
| Tetanus/diphtheria | X |  |  |  |  |  |  |  |  |  |  |  |  | X |  |
| IgG | X | X |  |  |  | X |  | X |  |  | X |  |  | X | X |
| IgM | X | X |  |  |  | X |  | X |  |  | X |  |  | X | X |
| IgA | X | X |  |  |  | X |  | X |  |  | X |  |  | X | X |
| IgE | X | X |  |  |  | X |  | X |  |  | X |  |  | X | X |
| IgG for 23 serotypes of strep pneumo | X | X |  |  |  | X |  | X |  |  | X |  |  | X | X |
| IgG for diphtheria | X | X |  |  |  | X |  | X |  |  | X |  |  | X | X |
| IgG for tetanus | X | X |  |  |  | X |  | X |  |  | X |  |  | X | X |
| T and B cell panel | X |  |  |  |  |  |  |  |  |  |  |  |  | X |  |

* Will be completed in women of child bearing age

**2. Introduction**

**2.1 Study Rationale**

Patients with CLL are at increased risk of infections as compared to age matched controls, with infections being a major cause of morbidity and mortality. Previous studies have shown that patients with CLL have both hypogammaglobinemia and impaired humoral immunity as defined by vaccine responses to both polysaccharide and peptide antigens. Attempts at decreasing infections in CLL have included therapy with prophylactic antibiotics and intravenous immunoglobulin. In general clinical practice and in previous studies, patients have started IV immunoglobulin replacement therapy if they have a history of serious infection or hypogammaglobinemia (defined as IgG below 400 g/dL), but vaccine responses have not been routinely evaluated. This study will provide a novel approach to identifying CLL patients with humoral immunodeficiency by checking both Ig levels and vaccine responses. In patients with impaired humoral immunity, we will use subcutaneous immunoglobulin replacement to show this intervention will be well tolerated and increase Ig levels and protective antibody titers.

**2.2 Background**

Lymphoproliferative disorders (LPD) are one of the most common hematologic malignancies. Each year in the U.S., approximately 15,000 patients are diagnosed with chronic lymphocytic leukemia (CLL). [1] Patients with CLL are at increased risk of infections as compared to age matched controls. [2] Furthermore, infections remain a major cause of morbidity and mortality, with up to 17% to 50% of infections being fatal. [2-4] Infection type and occurrence in CLL are related to disease stage and treatment history. CLL carries a known increased risk of bacterial infections, but certain anti-neoplastic agents increase the risk of viral infections, while the Bruton kinase inhibitor ibrutinib may decrease rates of infection. [5] Attempts at decreasing infections in CLL have included therapy with prophylactic antibiotics and intravenous immunoglobulin. In general clinical practice and in previous studies, patients have started immunoglobulin replacement therapy if they have a history of serious infection or hypogammaglobinemia (defined as IgG below 400 g/dL). [4-8] Immunoglobulin replacement therapy has been shown to decrease rates of bacterial infection in some studies, but the utility of the universal application of the practice remains unclear. A Cochrane meta-analysis concluded that the use of IVIG may be considered in patients with LPD with hypogammaglobinemia and recurrent infection, but acknowledged that the studies are heterogeneous. [9]

Previous studies have shown that patients with CLL have impaired humoral immunity as defined by vaccines responses to both polysaccharide and peptide antigens. [9] Patients with primary immunodeficiency are routinely evaluated for their humoral antibody response before immunoglobulin replacement is considered. However, the practice of checking vaccine responses prior to starting immunoglobulin replacement has been advocated, but not extensively studied in patients with CLL. [5, 10-12] Previous data has shown that low titers to pneumococcal vaccination was more highly associated with risk of infection as compared to serum IgG. [13] Thus, an immune evaluation including vaccine titers may help to identify CLL patients most at risk for life threatening infection as compared to patients with hypogammaglobinemia but preserved humoral immunity or patients with clinical infections alone with preserved immunoglobulin levels and function. Patients with impaired humoral immunity may benefit most from immunoglobulin prophylaxis.

Previous trials and clinical practice have used intravenous immunoglobulin in patients with CLL with recurrent infections and hypogammaglobinemia. [6, 8, 14] As compared to subcutaneous immunoglobulin, intravenous immunoglobulin carries a higher risk of an infusion reaction (including aseptic meningitis), renal dysfunction, theoretical risk of hypercoagulability, along with the need for trained medical personnel to provide intravenous access. [15, 16] Subcutaneous immunoglobulin mitigates these risks and can also be administered at home, providing much greater patient autonomy. [16-18] There is limited data on the use of SCIG in patients with secondary immunodeficiency and CLL, but one center has published experience with SCIG in lymphoproliferative diseases, including 21 patients with CLL. [19] A recent randomized study evaluated the use of SCIG in 24/46 patients with multiple myeloma, but did not detail the immune evaluation. [20]

This study is novel in that it will stratify patients with CLL according to their humoral response to peptide and polysaccharide vaccines, and will employ subcutaneous immunoglobulin replacement in those with an impaired humoral response.

**2.3 Risk/Benefit Assessment**

**2.3.1 Known Potential Risks**

- Immediate risks for the initial laboratory evaluation include the risks associated with phlebotomy (pain, bleeding, infection). For patients with humoral immunodeficiency who will be treated with subcutaneous Ig replacement with Hizentra, please see the package insert for potential risks.
- The investigators cannot identify any long term risks with this study beyond the risks associated with Hizentra, which are covered in the package insert.

**2.3.2 Known Potential Benefits**

- Immediate potential benefits include the identification of humoral immunodeficiency
- Long-term potential benefits include:
  - Decreased risk of non-neutropenic infection
  - Improved quality of life

**2.3.3 Assessment of Potential Risks and Benefits**

Subcutaneous Ig replacement therapy with Hizentra is generally well tolerated by patients with primary immunodeficiency, and generally has a better safety profile as compared to IV Ig replacement therapy. In the opinion of the investigators, the nominal risks of a laboratory evaluation followed by therapy with Hizentra are outweighed by the benefits of identification of humoral immunodeficiency in patients who otherwise remain undiagnosed. Identification of these patents will lead to therapy with subcutaneous Ig replacement, which may decrease the risk of non-neutropenic infection and potentially improve quality of life.

**3. Objectives and Endpoints**

1. The objective of this study is to identify patients with CLL who have serum IgG ≥400 mg/dL yet still have demonstrable humoral immunodeficiency.

To assess this objective, patients will undergo the following immune evaluation:

- Check serum IgG, IgM, IgA, IgE
- Check lymphocyte subsets, including CD4, CD8, CD3, and CD19
- Evaluate pre and post vaccine IgG responses 4 weeks apart to the following antigens: streptococcus pneumoniae, diphtheria, tetanus

Normal response to vaccination defined as follows:

- Strep pneumoniae (based on response to PPV)
  - If < 1.3, need to increase 2 fold to above 1.3 OR increase 4 fold
  - If > 1.3, need to increase 2 fold
  - Responses need to be demonstrated by 70% of serotypes
- Diphtheria, normal response
  - 2 fold increase into protective range
- Tetanus, normal response
  - 2 fold increase into protective range

Impaired vaccine response defined at abnormal response to any of the above antigens

2. Additional objectives of the study include to show that subcutaneous Ig replacement therapy is efficacious in increasing Ig levels and protective antibody titers in patients with CLL.

To assess this objective, IgG, IgM, IgA, IgE and IgG titers for streptococcus pneumoniae, diphtheria, tetanus will be checked periodically on therapy with subcutaneous Ig.

3. Another secondary objective will be to measure patient quality of life while on subcutaneous Ig replacement therapy.

To assess this objective, patients will complete SF-36 forms, which are a validated measure of patient quality of life.

**4. Study Design**

**4.1 Overall Design**

Hypothesis: By providing a novel approach to identifying CLL patients with humoral immunodeficiency by checking both Ig levels and vaccines responses., we will identify patients with impaired humoral immunity despite serum IgG ≥400 mg/d. In these patients with impaired humoral immunity, we will use subcutaneous immunoglobulin replacement to show this intervention will be well tolerated and increase Ig levels and protective antibody titers.

Phase of the trial: Phase 2

Trial Design: Single center, case series.

Methods used to minimize bias: Most of the endpoint are based on laboratory evaluation. The quality of life evaluation is a validated measure.

Dosing: Subcutaneous Ig replacement with Hizentra will be administered at 100 mg/kg/week, which is a typical dose used for Ig replacement in patients with immunodeficiency. The specific dose of Hizentra may be adjusted due to significant changes in weight during the study time period.

Study groups/arms: Patients with CLL will undergo a laboratory evaluation for impaired humoral function. All patients with an abnormal evaluation will all be offered therapy with subcutaneous Ig replacement. We plan to enroll 10 patients into the therapy portion of the study.

Single or multi-site study: Single.

Name of intervention: Hizentra

Interim analysis: An interim analysis is not planned at this time.

Study stratifications: There are no planned stratifications.

**4.2 Scientific Rationale for the Study**

Patients with CLL are at increased risk of infections as compared to age matched controls, with infections being a major cause of morbidity and mortality. Previous studies have shown that patients with CLL have both hypogammaglobinemia and impaired humoral immunity as defined by vaccine responses to both polysaccharide and peptide antigens. Attempts at decreasing infections in CLL have included therapy with prophylactic antibiotics and intravenous immunoglobulin. In general clinical practice and in previous studies, patients have started IV immunoglobulin replacement therapy if they have a history of serious infection or hypogammaglobinemia (defined as IgG below 400 g/dL). This study will provide a novel approach to identifying CLL patients with humoral immunodeficiency by checking both Ig levels and vaccines responses. In patients with impaired humoral immunity, we will use subcutaneous immunoglobulin replacement to show this intervention will be well tolerated and increase Ig levels and protective antibody titers. We hope this case series will lead to future studies with control groups and larger sample sizes.

**4.3 Justification for Dose**

Patients who qualify for subcutaneous Ig replacement therapy with Hizentra will receive a dose of 100 mg/kg/week, which is a typical dose used for Ig replacement in patients with immunodeficiency. As compared to primary immunodeficiency, patients with secondary immunodeficiency typically require lower doses of Ig replacement. A dose of 100 mg/kg/week falls on the lower end of the typical range for replacement dosing for Ig therapy.

**4.4 End of Study Definition**

A subject is considered to have completed the study if:

- They have a normal immune evaluation
- They have an abnormal immune evaluation and complete 6 months of therapy with subcutaneous Ig followed by the laboratory evaluation following a 3 month wash out period.

**5. Study Population**

**5.1 Inclusion Criteria**

In order to participate in this study, a subject must meet all of the following criteria:

- Diagnosis of chronic lymphocytic leukemia
- Medically stable, with expected survival of > 1 year
- Able to understand and willingness to sign a written informed consent
- Able to comply with study procedures

**5.2 Exclusion Criteria**

Any subject who meets any of the following criteria will be excluded from the study:

- Previously diagnosed primary immunodeficiency
- Additional immunosuppressive states as assessed by the primary or co-investigators
- Ongoing therapy with Ig replacement
- Serum IgG < 400 mg/dL

**5.3 Lifestyle considerations**

There are no restrictions pertaining to lifestyle or diet due to this study.

**5.4 Screen Failures**

Due to the nature of this study, there will be no screen failures. All patients will CLL will be evaluated, with patients with an abnormal immune evaluation going on to the therapy phase of the study with subcutaneous Ig replacement. Patients will a normal immune evaluation will not be offered therapy with subcutaneous Ig replacement, but will be included in the study as well.

**5.5 Strategies for Recruitment and Retention**

The study will largely recruit from the hematology/oncology division at Rochester Regional Health. The majority of active CLL patients are enrolled in a registry, which will aid in identifying potential subjects. The registry has roughly 300-350 patients with CLL. Additionally, through professional networks and relationships, the study will also be advertised to other local hematology/oncology practices, each of whom have contributed patients in the past to previous research studies. In total, we expect to evaluate 20-30 patients to have 10 qualify for the therapy portion of the study with subcutaneous Ig replacement.

Once enrolled, subjects will be in close contact with the investigators and the clinical research support staff regarding the results of their laboratory evaluation, and if applicable, the administration of subcutaneous Ig replacement. For subjects being treated with subcutaneous Ig replacement, the first 2-3 visits will be done on site with the clinical research coordinator. If and when subjects are comfortable self-administering subcutaneous Ig therapy at home, the clinical research coordinator will be in phone contact at fixed intervals regarding tolerability of therapy. Subjects will also be asked to pick up supplies for Ig replacement on a monthly basis from the clinical research coordinator.

**6. Study Intervention**

**6.1 Study Intervention Administration**

**6.1.1 Study Intervention Description**

Hizentra 100 mg/kg/week x 24 weeks

**6.1.2 Dosing and Administration**

Each subject will be treated with a fixed dose of subcutaneous Hizentra 100 mg/kg/week for 24 weeks. Hizentra will be administered at the study site until patients are comfortable with self-administration at home. Typically, this will take 2-3 sessions on site with the assistance of the clinical research coordinator. Once patients are self-administering at home, they can administer at any time of day. There will be no defined relationship to administration of Hizentra in regards to meals. All subjects will discontinue Hizentra after 24 weeks of therapy, and will subsequently monitored for a 3 month wash out period. The specific dose of Hizentra may be adjusted due to significant changes in weight during the study time period.

Subjects will be advised regarding the following points regarding administration:

- Prior to administration, visually inspect each vial of Hizentra for particulate matter or discoloration, whenever the solution and container permit.
- Do not freeze. Do not use any solution that has been frozen.
- Check the product expiration date on the vial label. Do not use beyond the expiration date.
- Do not mix Hizentra with other products.
- Do not shake the vial.
- Use aseptic technique when preparing and administering this product.
- The Hizentra vial is for single-use only. Discard all used administration supplies and any unused product immediately after each infusion in accordance with local requirements.

Subjects will be advised regarding the following points regarding dosing:

- Hizentra is intended for subcutaneous administration using an infusion pump.
- Infuse Hizentra in the abdomen, thigh, upper arm, and/or lateral hip.
- Infusion sites – A Hizentra dose may be infused into multiple infusion sites. Use up to 8 infusion sites in parallel. More than one infusion device can be used simultaneously. Infusion sites should be at least 2 inches apart. Change the actual site of infusion with each administration.
- Volume (as tolerated) *–* For the first infusion of Hizentra, do not exceed a volume of 15 mL per infusion site. For subsequent infusions, the volume may be increased to 25 mL per infusion site.
- Rate (as tolerated) *–* For the first infusion of Hizentra, the recommended flow rate is up to 15 mL per hour per infusion site. For subsequent infusions, the flow rate may be increased to 25 mL per hour.

**6.2 Preparation/Handling/Storage/Accountability**

**6.2.1 Acquisition and Accountability**

CSL Behring will ship an initial drug supply based upon an estimated patient weight (eg. 80 kg) for the first 2 study subjects using 20ml and 10 ml vial sizes. CSL Behring will work with the study site to determine the best vial sizes per study subject needs. For all remaining study subjects, the study site will be in close contact with CSL Behring to provide specific drug supply as needed.

NEED TO ASK PATTY regarding how we will receive Hizentra (how much at what time). Hizentra will be stored in a locked clinical research facility and will be dispensed to subjects once monthly once they are comfortable self-administering at home. Hizentra will be stored at room temperature for the entirety of its shelf life, up to 30 months.

**6.2.2 Formulation, Appearance, Packaging, and Labeling**

Hizentra will be dispensed to subjects once monthly and will be stored at room temperature.

**6.2.3 Product Storage and Stability**

Hizentra will be stored at room temperature for the entirety of the study.

**6.2.4 Preparation**

Hizentra will be infused from each vial with the assistance of an infusion pump per 6.1.2.

**6.3 Measures to Minimize Bias: Randomization and Blinding**

Given the study design: single center case series, there will be no randomization or blinding for the study.

**6.4 Study Intervention Compliance**

Adherence to the protocol will be monitored by regular communication between subjects and the investigators and clinical research coordinators. Reminder phone calls will be made to patients for timely lab testing, and patients will complete an SF 36 at defined study visits.

**6.5 Concomitant Therapy**

Subjects will be allowed to continue all concurrent medical therapies during the entirety of the study. As per the exclusion criteria, Ig replacement from outside the study is not allowed at any time. Subjects will also be asked to report the use of any antibiotic courses during the study.

**6.5.1 Rescue Medication**

The study site will not be providing any rescue medications for the purposes of this study.

**7. Study intervention discontinuation and participant discontinuation/withdrawal**

**7.1 Discontinuation of Study Intervention**

Study intervention will also halt if patients experience a hypersensitivity reaction to the study drug. The study intervention will also be halted if there is any serious adverse event deemed related to the study drug by the study investigators. If the study intervention is halted, then pre and post vaccination titers will be obtained after subcutaneous immunoglobulin has been discontinued for 3 months. Infections will continue to be tracked through patient contact, the electronic medical record, and pharmacy data. Quality of life data will still be tracked through patient contact.

**7.2 Participant Discontinuation/Withdrawal from the Study**

The study intervention will be discontinued at any time a patient wishes to withdraw for any reason. Subjects will continue with medical care as prior to study enrollment.

All efforts will be made to continue to follow patients even if they are no longer receiving study drug. Study subjects may be withdrawn at any time based on the discretion of the investigator for safety, behavioral, or administrative reasons (including, but not limited to, an adverse event, lymphoma progression, protocol deviation, loss to follow up, non-adherence, or study termination).

In accordance with International Conference on Harmonisation (ICH) principles of Good Clinical Practice (GCP) the investigator always has the option to advise a subject to withdraw from the study over concerns of safety or subject well-being.

**7.3 Lost to Follow-Up**

A participant will be considered lost to follow-up if he or she fails to return for scheduled visits and is unable to be contacted by the study staff. If a participant fails to return to the clinic for a required study visit, the site will attempt to contact the participant and reschedule the missed visit, counsel the participant on the importance of maintaining the assigned visit schedule, and ascertain if the participant wishes to continue in the study.

Before a participant is deemed lost to follow-up, the investigator or designee will make every effort to regain contact with the participant, including 3 telephone calls and, if necessary, a certified letter to the participant’s last known mailing address. Should the participant continue to be unreachable, he or she will be considered to have withdrawn from the study with a primary reason of lost to follow-up

**8. Study Assessments and Procedures**

**8.1 Efficacy Assessments**

Efficacy will be assessed by the study drug increasing immunoglobulin levels, tetanus, diphtheria, and pneumococcal IgG levels, and study drug safety and tolerability in this population.

The secondary endpoint of quality of life will be assessed by the SF-36 quality of life assessment (please see attachment), which is a validated questionnaire. This will be administered monthly by the study coordinator.

The secondary endpoint of the frequency of humoral deficiency in patients with chronic lymphocytic leukemia will be assessed with the following laboratory data:

- IgG, IgM, IgA, IgE, CD4, CD8, CD3, and CD19 lymphocyte subsets
- Pre and post vaccine IgG responses 4 weeks apart to the following antigens: *Streptococcus pneumoniae*, diphtheria, tetanus, with a normal response defined as followed:
  - Strep pneumoniae (based on response to PPV):
    - If < 1.3, need to increase 2 fold to above 1.3 OR increase 4 fold
    - If > 1.3, need to increase 2 fold
    - Responses need to be demonstrated by 70% of serotypes
  - Diphtheria, normal response
    - 2 fold increase into protective range
  - Tetanus, normal response
    - 2 fold increase into protective range

Impaired vaccine response defined at abnormal response to any of the above antigens.

All blood specimens will be handled processed at the Rochester Regional Health laboratories as per usual clinical care. Laboratory data will be provided and explained to study subjects upon request.

**8.2 Safety and Other Assessments**

Safety will be assessed by all SAEs that will be tracked monthly. SAEs will be managed by study investigators and/or subjects’ other medical practitioners per usual clinical practice.

Subjects will be asked about any injection site reactions at each visit. Vital signs (height, weight, blood pressure, pulse) and physical examination (general appearance, HEENT exam, cardiovascular exam, pulmonary exam, extremities, gross neurological status, cutaneous exam) will be assessed at each study visit.

**8.3 Adverse Events and Serious Adverse Events**

**8.3.1 Definition of Adverse Events**

An adverse event means any untoward medical occurrence associated with the use of an intervention in humans, whether or not considered intervention-related.

**8.3.2 Definition of Serious Adverse Event**

A Serious adverse event (SAE) is considered "serious" if, in the view of either the investigator or sponsor, it results in any of the following outcomes: death, a life-threatening adverse event, inpatient hospitalization or prolongation of existing hospitalization, a persistent or significant incapacity or substantial disruption of the ability to conduct normal life functions, or a congenital anomaly/birth defect.

Important medical events that may not result in death, be life-threatening, or require hospitalization may be considered serious when, based upon appropriate medical judgment, they may jeopardize the participant and may require medical or surgical intervention to prevent one of the outcomes listed in this definition.

**8.3.3 Classification of an Adverse Event**

**8.3.3.1 Severity of Event**

The following guidelines will be used to describe severity

• Mild – Events require minimal or no treatment and do not interfere with the participant’s daily activities.

• Moderate – Events result in a low level of inconvenience or concern with the therapeutic measures. Moderate events may cause some interference with functioning.

• Severe – Events interrupt a participant’s usual daily activity and may require systemic drug therapy or other treatment. Severe events are usually potentially life-threatening or incapacitating.

**8.3.3.2 Relationship to Study Intervention**

The investigator will assess the relationship between the AE and the study drug. All AEs will be classified as either related or not related to the study drug.

Related: The AE is known to occur with the study intervention, there is a reasonable possibility that the study intervention caused the AE, or there is a temporal relationship between the study intervention and event. Reasonable possibility means that there is evidence to suggest a causal relationship between the study intervention and the AE.

Not Related – There is not a reasonable possibility that the administration of the study intervention caused the event, there is no temporal relationship between the study intervention and event onset, or an alternate etiology has been established.

**8.3.3.3 Expectedness**

The study investigators will be responsible for determining whether an adverse event (AE) is expected or unexpected. An AE will be considered unexpected if the nature, severity, or frequency of the event is not consistent with the risk information previously described for the study intervention

**8.3.4 Time Period and Frequency for Event Assessment and Follow-Up**

The occurrence of an adverse event (AE) or serious adverse event (SAE) may come to the attention of study personnel during study visits and interviews of a study participant presenting for medical care, or upon review by a study monitor. All AEs including local and systemic reactions not meeting the criteria for SAEs will be captured on the appropriate case report form (CRF). Information to be collected includes event description, time of onset, clinician’s assessment of severity, relationship to study product (assessed only by those with the training and authority to make a diagnosis), and time of resolution/stabilization of the event. All AEs will be followed to adequate resolution. Any medical condition that is present at the time that the participant is screened will be considered as baseline and not reported as an AE. However, if the study participant’s condition deteriorates at any time during the study, it will be recorded as an AE. Changes in the severity of an AE will be documented to allow an assessment of the duration of the event at each level of severity to be performed.

All SAEs and AEs will start at the time of giving written informed consent for participation in the current study and finish with the end of study visit.

**8.3.5 Adverse Event Reporting**

At each clinical evaluation, the investigator or delegate will determine if any AEs have occurred. These will be recorded in a case report form. A medical diagnosis will be written if known. The investigator will follow the course of the AE.

**8.3.6 Serious Adverse Event Reporting**

The study clinician will immediately report to the sponsor any serious adverse event, whether or not considered study intervention related, including those listed in the protocol or investigator brochure and will include an assessment of whether there is a reasonable possibility that the study intervention caused the event. This report will occur within 24 hours of the investigator becoming aware of the event.

All serious adverse events (SAEs) will be followed until satisfactory resolution or until the site investigator deems the event to be chronic or the participant is stable.

**8.3.7 Reporting Events to Participants**

Subjects will be informed of adverse events if they are deemed related to the study drug and are unanticipated based on the study drug profile.

**8.3.8 Events of Special Interest**

Not applicable

**8.3.9 Reporting of Pregnancy**

Not applicable given the targeted study population

**8.4 Unanticipated Problems**

**8.4.1 Definition of Unanticipated Problems**

The Office for Human Research Protections (OHRP) considers unanticipated problems involving risks to participants or others to include, in general, any incident, experience, or outcome that meets all of the following criteria:

• Unexpected in terms of nature, severity, or frequency given (a) the research procedures that are described in the protocol-related documents, such as the Institutional Review Board (IRB)- approved research protocol and informed consent document; and (b) the characteristics of the participant population being studied

• Suggests that the research places participants or others at a greater risk of harm (including physical, psychological, economic, or social harm) than was previously known or recognized.

**8.4.2 Unanticipated Problem Reporting**

The investigator will report unanticipated problems to the reviewing Institutional Review Board (IRB). The UP report will include the following information:

• Protocol identifying information: protocol title and number, PI’s name, and the IRB project number

• A detailed description of the event, incident, experience, or outcome;

• An explanation of the basis for determining that the event, incident, experience, or outcome represents an UP

• A description of any changes to the protocol or other corrective actions that have been taken or are proposed in response to the UP.

UPs that are serious adverse events (SAEs) will be reported to the IRB and to the study sponsor within of the investigator becoming aware of the event. • Any other UP will be reported to the IRB and the study sponsor within of the investigator becoming aware of the problem.

**8.4.3 Reporting Unanticipated Problems to Participants**

The study investigators will report unanticipated problems to all study subjects.

**9. Statistical Considerations**

**9.1 Statistical Hypotheses**

Patients with CLL will demonstrate humoral immunodeficiency despite serum IgG ≥400 mg/dL

Secondary Hypotheses

- Subcutaneous Ig replacement therapy in increasing Ig levels
- Subcutaneous Ig replacement therapy will provide protective antibody titers for diphtheria, tetanus, and streptococcus pneumoniae
- Subcutaneous Ig replacement therapy will be safe and tolerable
- Quality of life (SF 36) will improve on subcutaneous Ig replacement therapy.

**9.2 Sample Size Determination**

There will be 10 subjects enrolled in this non-randomized, proof of concept study

**9.3 Populations for Analyses**

This will be a per protocol analysis dataset.

**9.4 Statistical Analyses**

**9.4.1 General Approach**

Descriptive statistics will be used for each endpoint

**9.4.2 Analysis of the Primary Efficacy Endpoint**

This will be analyzed using descriptive statistics

**9.4.3 Analysis of the Secondary Endpoint(S)**

These will be analyzed using descriptive statistics

**9.4.4 Safety Analyses**

The safety analyses will be approached through descriptive statistics

**9.4.5 Tabulation of Individual Participant Data**

Individual participant data may be presented

**10. Supporting Documentation and Operational Considerations**

**10.1 Regulatory, Ethical, and Study Oversight Considerations**

**10.1.1 Informed Consent Process**

**10.1.1.1 Consent/Assent and Other Informational Documents Provided To Participants**

Consent forms describing in detail the study intervention, study procedures, and risks are given to the participant and written documentation of informed consent is required prior to starting intervention/administering study intervention. The consent form is submitted with this protocol.

**10.1.1.2 Consent Procedures and Documentation**

Informed consent is a process that is initiated prior to the individual’s agreeing to participate in the study and continues throughout the individual’s study participation. Consent forms will be Institutional Review Board (IRB)-approved and the participant will be asked to read and review the document. The investigator will explain the research study to the participant and answer any questions that may arise. A verbal explanation will be provided in terms suited to the participant’s comprehension of the purposes, procedures, and potential risks of the study and of their rights as research participants. Participants will have the opportunity to carefully review the written consent form and ask questions prior to signing. The participants should have the opportunity to discuss the study with their family or surrogates or think about it prior to agreeing to participate. The participant will sign the informed consent document prior to any procedures being done specifically for the study. Participants must be informed that participation is voluntary and that they may withdraw from the study at any time, without prejudice. A copy of the informed consent document will be given to the participants for their records. The informed consent process will be conducted and documented in the source document (including the date), and the form signed, before the participant undergoes any study-specific procedures. The rights and welfare of the participants will be protected by emphasizing to them that the quality of their medical care will not be adversely affected if they decline to participate in this study

**10.1.2 Study Discontinuation and Closure**

This study may be temporarily suspended or prematurely terminated if there is sufficient reasonable cause. If the study is prematurely terminated or suspended, the Principal Investigator (PI) will promptly inform study participants, the Institutional Review Board (IRB), and sponsor and will provide the reason(s) for the termination or suspension. Study participants will be contacted, as applicable, and be informed of changes to study visit schedule. Circumstances that may warrant termination or suspension include, but are not limited to:

• Determination of unexpected, significant, or unacceptable risk to participants

• Demonstration of efficacy that would warrant stopping

• Insufficient compliance to protocol requirements

• Data that is not sufficiently complete and/or evaluable

• Determination that the primary endpoint has been met

• Determination of futility

**10.1.3 Confidentiality and Privacy**

All study subject names and contact information will be kept confidential. Subjects will be allotted a number during participation. Only study personnel will have access to patient contact information, and this will be kept in a password-protected file. All research activities will be conducted in as private a setting as possible. Authorized representatives of the sponsor, representatives of the Institutional Review Board (IRB), regulatory agencies or pharmaceutical company supplying study product may inspect all documents and records required to be maintained by the investigator, including but not limited to, medical records (office, clinic, or hospital) and pharmacy records for the participants in this study. The clinical study site will permit access to such records.

**10.1.4 Future Use of Stored Specimens and Data**

Data collected for this study will be analyzed and stored at the Allergy/Immunology office at Rochester Regional Health. After the study is completed, the de-identified, archived data will be transmitted to and stored on the Rochester Regional Health server in a password protected file. There will be no biologic specimens collected. There will be no genetic testing.

**10.1.5 Key Roles and Study Governance**

**Principal Investigator:**

S. Shahzad Mustafa, MD

Allergy/Immunology, Rochester Regional Health

Clinical Assistant Professor of Medicine, University of Rochester

222 Alexander Street, Suite 3000 Rochester, NY 14607

**Co-Investigator:**

Allison Ramsey, MD

Allergy/Immunology, Rochester Regional Health

Clinical Assistant Professor of Medicine, University of Rochester

222 Alexander Street, Suite 3000 Rochester, NY 14607

**Co-Investigator:**

Saad Jamshed, MD

Hematology/Oncology, Rochester Regional Health

20 Hagen Drive #100

Rochester, NY 14625

**Study Coordinator:**

Dawn Sheflin, RN

222 Alexander Street, Suite 3000 Rochester, NY 14607

**10.1.6 Quality Assurance and Quality**

Our site will be responsible for internal quality management of study conduct, data and biological specimen collection, documentation and completion. The study will be in compliance with the protocol, International Conference on Harmonisation Good Clinical Practice (ICH GCP), and applicable regulatory requirements (e.g., Good Laboratory Practices (GLP), Good Manufacturing Practices (GMP)). The investigational site will provide direct access to all trial related sites, source data/documents, and reports for the purpose of monitoring and auditing by the sponsor, and inspection by local and regulatory authorities.

**10.1.7 Data Handling and Record Keeping**

**10.1.7.1 Data Collection and Management Responsibilities**

Source data will include patient interviews, hospital records, clinical and office EMRs, and laboratory data. Study participation will be captured in a participant’s medical record. Data collection is the responsibility of the clinical trial staff at the site under the supervision of the site investigator. The investigator is responsible for ensuring the accuracy, completeness, legibility, and timeliness of the data reported. All source documents will be completed in a neat, legible manner to ensure accurate interpretation of data. Hardcopies of the study visit worksheets will be provided for use as source document worksheets for recording data for each participant enrolled in the study. Clinical data (including adverse events (AEs), concomitant medications, and expected adverse reactions data) and clinical laboratory data will be entered into a password protected Excel spreadsheet. Clinical data will be entered directly from the source documents.

**10.1.7.2 Study Records Retention**

Study documents will be retained for a minimum of 2 years after the last approval of a marketing application in an International Conference on Harminosation (ICH) region

**10.1.8 Protocol Deviations**

A protocol deviation is any noncompliance with the clinical trial protocol, International Conference on Harmonisation Good Clinical Practice (ICH GCP), or Manual of Procedures (MOP) requirements. The noncompliance may be either on the part of the participant, the investigator, or the study site staff. As a result of deviations, corrective actions will be developed by the site and implemented promptly. These practices are consistent with ICH GCP. The site investigator will identify and report deviations within 2 working days of identification of the protocol deviation, or within working days of the scheduled protocol-required activity. Protocol deviations will be sent to the reviewing Institutional Review Board (IRB) per their policies.

**10.1.9 Publication And Data Sharing Policy**

This trial will be registered at ClinicalTrials.gov, and results information from this trial will be submitted to ClinicalTrials.gov. In addition, every attempt will be made to publish results in peer reviewed journals. Data from this study may be requested from other researchers 0.5 years after the completion of the primary endpoint by contacting S. Shahzad Mustafa, MD.

**10.1.10 Conflict of Interest Policy**

Any perceived or real conflict of interest will be disclosed and managed. All study group members will disclose all conflicts of interest and will establish a mechanism for the management of all reported dualities of interest
