# Supplementary material for: Subcutaneous immunoglobulin replacement for treatment of humoral immune dysfunction in patients with chronic lymphocytic leukemia
Source: PLoS One. 2021 Oct 15;16(10):e0258529. doi: 10.1371/journal.pone.0258529 (PMC8519417; doi:10.1371/journal.pone.0258529)
Supplement: S3 File — (DOCX) [file pone.0258529.s004.docx]

Figure 1:

IgG Comparison of baseline vs 3 months

. signrank baseline = months

Wilcoxon signed-rank test

sign | obs sum ranks expected

-------------+---------------------------------

positive | 0 0 22.5

negative | 9 45 22.5

zero | 0 0 0

-------------+---------------------------------

all | 9 45 45

unadjusted variance 71.25

adjustment for ties 0.00

adjustment for zeros 0.00

----------

adjusted variance 71.25

Ho: baseline = months

z = -2.666

Prob > |z| = 0.0077

IgG comparison baseline vs 3 months after last dose

. signrank baseline = monthsafterlastdose

Wilcoxon signed-rank test

sign | obs sum ranks expected

-------------+---------------------------------

positive | 3 10 22.5

negative | 6 35 22.5

zero | 0 0 0

-------------+---------------------------------

all | 9 45 45

unadjusted variance 71.25

adjustment for ties 0.00

adjustment for zeros 0.00

----------

adjusted variance 71.25

Ho: baseline = monthsafterlastdose

z = -1.481

Prob > |z| = 0.1386

IgG comparison baseline vs 6 months

. signrank baseline = var3

Wilcoxon signed-rank test

sign | obs sum ranks expected

-------------+---------------------------------

positive | 0 0 22.5

negative | 9 45 22.5

zero | 0 0 0

-------------+---------------------------------

all | 9 45 45

unadjusted variance 71.25

adjustment for ties 0.00

adjustment for zeros 0.00

----------

adjusted variance 71.25

Ho: baseline = var3

z = -2.666

Prob > |z| = 0.0077

- Strep IgG basleine vs 3 months

. signrank baseline = months

Wilcoxon signed-rank test

sign | obs sum ranks expected

-------------+---------------------------------

positive | 0 0 22.5

negative | 9 45 22.5

zero | 0 0 0

-------------+---------------------------------

all | 9 45 45

unadjusted variance 71.25

adjustment for ties -0.25

adjustment for zeros 0.00

----------

adjusted variance 71.00

Ho: baseline = months

z = -2.670

Prob > |z| = 0.0076

- Strep IgG level baseline vs 6 months
- . signrank baseline = var4
- Wilcoxon signed-rank test
- sign | obs sum ranks expected
- -------------+---------------------------------
- positive | 0 0 22.5
- negative | 9 45 22.5
- zero | 0 0 0
- -------------+---------------------------------
- all | 9 45 45
- unadjusted variance 71.25
- adjustment for ties -0.25
- adjustment for zeros 0.00
- ----------
- adjusted variance 71.00
- Ho: baseline = var4
- z = -2.670
- Prob > |z| = 0.0076
- . signrank baseline = 3monthsafterlastdose
- Wilcoxon signed-rank test
- sign | obs sum ranks expected
- -------------+---------------------------------
- positive | 1 6 19.5
- negative | 5 33 19.5
- zero | 3 6 6
- -------------+---------------------------------
- all | 9 45 45
- unadjusted variance 71.25
- adjustment for ties -0.50
- adjustment for zeros -3.50
- ----------
- adjusted variance 67.25
- Ho: baseline = monthsafterlastdose
- z = -1.646
- Prob > |z| = 0.0997

6/29/21

Reviewers asked for CI instead of P value

For Ig baseline and at 6 months

. ci means baseline signdiff var4 absdiff

Variable | Obs Mean Std. Err. [95% Conf. Interval]

-------------+---------------------------------------------------------------

baseline | 9 676.1111 65.18935 525.7842 826.438

signdiff | 9 1 0 1 1

var4 | 9 1081.889 51.46667 963.2065 1200.571

absdiff | 9 405.7778 58.28945 271.3621 540.1935

we reported absdiff

for strep pneu

. ci means baseline var4 absdiff

Variable | Obs Mean Std. Err. [95% Conf. Interval]

-------------+---------------------------------------------------------------

baseline | 9 6.666667 1.354006 3.544322 9.789011

var4 | 9 17 1.301708 13.99826 20.00174

absdiff | 9 10.33333 1.47196 6.938987 13.72768

Ig G baseline vs 1, 3, 6, after 4 weeks

. ci means baseline absdiff absdiff1 absdiff2 absdiff3

Variable | Obs Mean Std. Err. [95% Conf. Interval]

-------------+---------------------------------------------------------------

baseline | 9 676.1111 65.18935 525.7842 826.438

absdiff | 9 223.1111 29.94785 154.0513 292.171

absdiff1 | 9 375.8889 24.46508 319.4723 432.3055

absdiff2 | 9 405.7778 58.28945 271.3621 540.1935

absdiff3 | 9 55 14.35367 21.90038 88.09962

For tetanus

. ci means baseline absdiff absdiff1 absdiff2 absdiff3

Variable | Obs Mean Std. Err. [95% Conf. Interval]

-------------+---------------------------------------------------------------

baseline | 9 1.117778 .1768221 .7100253 1.52553

absdiff | 9 1.302222 .3784317 .4295573 2.174887

absdiff1 | 9 2.307778 .591638 .9434581 3.672097

absdiff2 | 9 1.248889 .1759928 .8430488 1.654729

absdiff3 | 9 .6388889 .1944754 .1904278 1.08735

For diptheria

. ci means baseline absdiff absdiff1 absdiff2 absdiff3

Variable | Obs Mean Std. Err. [95% Conf. Interval]

-------------+---------------------------------------------------------------

baseline | 9 .2177778 .0665369 .0643434 .3712122

absdiff | 9 .4133333 .256293 -.1776794 1.004346

absdiff1 | 9 .77 .347463 -.0312512 1.571251

absdiff2 | 9 .5444445 .2398849 -.0087311 1.09762

absdiff3 | 9 .2544445 .1465256 -.0834442 .5923331

for strep

. ci means baseline absdiff absdiff1 absdiff2 absdiff3

Variable | Obs Mean Std. Err. [95% Conf. Interval]

-------------+---------------------------------------------------------------

baseline | 9 6.666667 1.354006 3.544322 9.789011

absdiff | 9 4.444444 .8012336 2.596796 6.292092

absdiff1 | 9 6.555556 .9444444 4.377663 8.733448

absdiff2 | 9 10.33333 1.47196 6.938987 13.72768

absdiff3 | 9 1.666667 .5527708 .3919749 2.941358
